# Hearing loss and cognition in the mid-to-late life transition

**DOI:** 10.1093/geroni/igaf122.3784

**Published:** 2025-12-31

**Authors:** Willa Brenowitz, Pamela Schreiner, Kathryn Hand, Paige Wartko, John Dickerson, Kelly Reavis, Kristine Yaffe

**Affiliations:** Kaiser Permanente, Portland, Oregon, United States; University of Minnesota, Minneapolis, Minnesota, United States; Oregon Health and Science University, Portland, Oregon, United States; Kaiser Permanente Washington Health Research Institute, Seattle, Washington, United States; Kaiser Permanente Center for Health Research, Portland, Oregon, United States; VA Portland Health Care System, Portland, Oregon, United States; University of California, San Francisco, San Francisco, California, United States

## Abstract

Hearing loss is associated with cognitive decline in later life, but few studies have established whether this association emerges earlier in midlife. We evaluated the cross-sectional association between audiometric hearing loss and cognition in individuals spanning ages 53-65 years. We studied 1,486 participants in the Coronary Artery Risk Development in Young Adults (CARDIA) study at a mean age of 61.2 (SD: 3.6), with 58.4% female, 46.4% Black, and 53.6% White. Hearing loss was defined as a pure tone average (PTA) threshold ≥25dB for speech (500-4000 Hz) and high (4000-8000 Hz) frequencies in the better hearing ear. Cognition was measured with the Montreal Cognitive Assessment (MoCA), digit symbol substitution test (DSST), and the Rey Auditory Verbal Learning Test delay recall score (RAVLT). Linear regression models estimated mean differences and 95% confidence intervals (CIs) for the associations between hearing loss in each frequency range and separate cognitive tests, adjusting for age, sex, education, race, health care access, diabetes, prevalent cardiovascular disease, stroke, hypertension, depression, body mass index, and smoking. Hearing loss (11% prevalence) in the speech detection range was associated with lower cognition in all tests (MoCA: -1.31 [95% CI:-1.81,-0.82]; DSST: -2.59 [-5.05,-0.14]; RAVLT: -0.69 [-1.23,-0.15]). High frequency hearing loss (43% prevalence) was also associated with lower cognition in (MoCA: -0.85 [-1.18, -0.52]; DSST: -2.82 [-4.43, -1.23]; and borderline associated with RAVLT: -0.32 [-0.67, 0.03]). Most hearing loss was mild. These findings in a population-based sample suggest that the associations between hearing loss and cognition may be detectable by midlife.

